# 儿童异基因造血干细胞移植后BK病毒脑炎临床研究

**DOI:** 10.3760/cma.j.issn.0253-2727.2021.10.005

**Published:** 2021-10

**Authors:** 娜 李, 晓军 黄, 昱 王, 盼 锁, 兰平 许, 开彦 刘, 晓辉 张, 晨华 闫, 峰蓉 王, 军 孔, 翼飞 程

**Affiliations:** 1 北京大学人民医院 100044 Peking University People's Hospital, Beijing 100044, China; 2 邢台市人民医院 054000 Xingtai People's Hospital, Xingtai 054000, China

**Keywords:** 异基因造血干细胞移植, BK病毒, 中枢神经系统感染, Allogeneic hematopoietic stem cell transplantation, BK virus, Central nervous system infection

## Abstract

**目的:**

探讨异基因造血干细胞移植患儿中BK病毒（BKV）脑炎的发病率、病死率、中位发病时间、临床表现、诊治及转归等，以提高临床医师对本病的认识。

**方法:**

回顾性分析2015年1月1日至2020年12月31日在北京大学人民医院接受单倍型造血干细胞移植治疗的709例儿童患者，其中14例诊断为BKV脑炎，分析其临床特征、治疗过程及转归。

**结果:**

BKV脑炎发生率为1.97％（14例）。患儿多为男性（12例），中位年龄为11岁，中位发病时间为移植后第55天。最常见的临床表现为意识障碍、抽搐发作（7例）。14例患儿予阿昔洛韦、更昔洛韦单用，或联合丙种球蛋白治疗，9例患儿痊愈，1例患儿死于病毒性脑炎，4例患儿死于其他疾病，病死率为35.7％。

**结论:**

BKV脑炎主要表现为脑炎或脑膜炎。虽然确诊BKV脑炎后积极予药物治疗，但许多患者仍死于多器官衰竭或其他并发症。当异基因造血干细胞移植患者出现神经系统症状、出血性膀胱炎时，必须高度警惕BKV脑炎，尽早施救，从而改善患者的生存率及生活质量。

## Abstract (en)

**Objective:**

To explore the morbidity, mortality, median onset time, clinical characteristics, diagnosis, treatment, and outcome of BK virus (BKV) central nervous system infection in children with allogeneic hematopoietic stem cell transplantation (allo-HSCT), and improve the understanding, clinical diagnosis, and treatment of the disease.

**Methods:**

Seven hundred and nine children who received haploid HSCT treatment in Peking University People's Hospital from January 1, 2015 to December 31, 2020 were reviewed. Fourteen patients were diagnosed with BKV central nervous system infection, and their clinical characteristics，treatment process, and prognosis were analyzed.

**Results:**

The incidence of BKV central nervous system infection was 1.97％ (14 cases), mostly in men (12 cases), with a median age of 11 years old and median onset time of 55 d. Additionally, most of the cases showed disturbance of consciousness and seizures (seven cases). Furthermore, 14 cases were treated with acyclovir and ganciclovir alone or with gamma globulin. Nine cases were cured, of which one died of viral encephalitis and four of other diseases, with a mortality rate of 35.7％.

**Conclusion:**

Individuals with central nervous system involvement by BKV infection, usually show signs and symptoms of acute encephalitis, with some cases being accompanied by meningeal involvement. Although BKV encephalitis was diagnosed and actively treated with drugs, many patients still died of multiple organ failure or other complications. Therefore, when there are neurological symptoms and hemorrhagic cystitis in patients with allo-HSCT, it is necessary to be highly vigilant against BKV central nervous system infection. This helps to make clear diagnosis and treatment quickly; thus, improving the survival rate and quality of life of patients with HSCT.

异基因造血干细胞移植（allo-HSCT）是治疗儿童血液病、肿瘤、免疫缺陷性疾病及遗传性疾病的有效手段[1]–[2]。然而，强烈的免疫抑制会引起诸多并发症，其中0.8％～15％的患者可能合并中枢神经系统感染，其病原体包括细菌、真菌、病毒和原生动物等，其预后差，病死率高[3]–[6]。而病毒是allo-HSCT后脑炎常见的病原体。

BK病毒（BK virus，BKV）系多瘤病毒科多瘤病毒属，是进行性多灶性白质脑病（PML）的病因。BKV在幼儿期血清阳性率可达60％～100％，在原发感染发生后，病毒可以在肾脏、肺、眼睛、肝脏、大脑等多部位潜伏，当机体免疫功能受到抑制时可重新激活。在高达50％的allo-HSCT患者中可发现BKV复制的证据，10％～25％的患者可出现BKV感染后出血性膀胱炎，而BKV相关脑炎的报道极少，且无儿童相关病例报道，因此，我们回顾性分析2015年1月1日至2020年12月31日于北京大学人民医院接受单倍型HSCT治疗的709例儿童患者，总结BKV脑炎的发病率、中位发病时间、临床表现及病死率等。

## 病例与方法

1. 病例：从2015年1月1日至2020年12月31日共有709例≤18岁的儿童（女255例，男454例）在北京大学人民医院接受了单倍型allo-HSCT治疗。其中急性和慢性血液系统恶性肿瘤542例（76.4％），骨髓衰竭或贫血138例（19.5％），实体器官肿瘤18例（2.5％），二次移植11例（1.6％）。

2. 诊断： BKV脑炎的临床表现及诊断尚无明确标准，但脑活检组织显示BKV DNA PCR阳性、免疫组织化学阳性或电子显微镜显示病毒颗粒为有效的诊断标准。由于病理组织活检的局限性，且没有关于BKV脑炎特异性的影像学特征的报道，临床诊断仍依赖于脑脊液中BKV DNA PCR阳性。本研究中BKV脑炎的诊断包括纳入标准和排除标准。纳入标准：①存在神经系统症状或体征；②脑活检组织或脑脊液中BKV DNA PCR阳性；③影像学改变为炎性改变或正常图像。排除标准：除外其他原因引起的细菌和真菌感染或脑病，如创伤、代谢紊乱、肿瘤和其他非传染性原因[7]。

3. 移植方案：供者的选择和移植方案与既往报道一致[8]–[9]。所有患者均接受外周血造血干细胞或骨髓加外周血造血干细胞的移植[8]，[10]。

4. 病毒感染预防：预处理期间予更昔洛韦预防病毒感染，回输造血干细胞当天至移植后1年予阿昔洛韦预防病毒感染。

5. 血液和脑脊液中的病毒监测：采用实时定量PCR检测EB病毒（EBV）和巨细胞病毒（CMV），并采用定性PCR检测其他病毒。在移植后前3个月每周检测2次EBV和CMV。对除EBV和CMV以外的病毒没有进行常规监测，只对可疑患者进行检测。

## 结果

1. 临床特征：通过脑脊液BKV DNA PCR检测阳性，且排除中枢神经系统白血病、血栓性微血管病（TMA）、药物相关脑病、细菌感染等疾病，我们确诊14例BKV脑炎患者，其发生率为1.97％。14例患儿临床特征见表1，其中包括12例男性和2例女性。患儿均有发热、意识障碍、抽搐等不同临床表现。原发病包括急性髓系白血病4例，急性淋巴细胞白血病6例，再生障碍性贫血2例，骨髓增生异常综合征2例。发病中位年龄为11（6～17）岁。从移植之日到出现症状的中位时间为55（16～296）d。其中4例患儿合并出血性膀胱炎。

**表1:** 14例异基因造血干细胞移植后合并BK病毒（BKV）脑炎患者临床特点、影像学表现、治疗及转归

例号	性别	年龄（岁）	原发病	症状	CSF PCR	血BKV PCR	尿BKV PCR	CSF白细胞（/µl）	CSF蛋白质（g/L）	影像学表现	合并其他病毒	发病时间	治疗用药	转归
1	男	12	AML	发热、头痛，计算力下降	BKV+	未查	阳性	0	0.52↑	MR：双侧顶枕叶异常信号，考虑可逆性后脑病综合征	CMV、EBV、腺病毒	+76d	更昔洛韦丙球	死亡
2	女	12	ALL	发热、头痛，恶心呕吐	BKV+	未查	阴性	10	1.82↑	CT：未见异常	无	+296d	更昔洛韦丙球	死亡
3	男	10	AML	意识不清、肢体抖动	BKV+	未查	阳性	0	正常	MR：双侧顶枕颞叶皮层下多发异常信号，考虑可逆性后脑病综合征	无	+26d	更昔洛韦丙球	痊愈
4	男	7	AML	发热	BKV+	未查	阳性	0	正常	MR：左侧胼胝体体部脱髓鞘病变，脑白质病/脱髓鞘病灶	CMV	+19d	阿昔洛韦	痊愈
5	女	6	SAA	双眼凝视、呼之不应	BKV+	阴性	阴性	0	正常	CT：未见异常	无	+17d	阿昔洛韦	死亡
6	男	7	SAA	发热、精神欠佳	BKV+	未查	阴性	0	0.11↓	CT：双侧脑室稍宽（同前）	无	+18d	阿昔洛韦	死亡
7	男	10	ALL	定向障碍、抽搐	BKV+	未查	阴性	18	0.94↑	MR：双侧海马结构、双侧大脑中央沟两旁脑回异常信号	无	+16d	更昔洛韦	痊愈
8	男	15	AML	抽搐	BKV+	阳性	阴性	42	0.66↑	CT：未见异常	CMV、EBV	+90d	更昔洛韦	痊愈
9	男	7	ALL	意识不清、昏迷、抽搐	BKV+	阳性	阴性	0	正常	CT：双侧侧脑室旁脱髓鞘病变	无	+50d	阿昔洛韦	死亡
10	男	8	ALL	口唇震颤	BKV+	未查	阴性	200	正常	MR：轻度脑萎缩（同前）	无	+60d	更昔洛韦	痊愈
11	男	16	ALL	间断妄想	BKV+	未查	阴性	0	正常	MR：左侧卵圆中心腔隙灶可能（同前）	无	+100d	阿昔洛韦	痊愈
12	男	17	ALL	面瘫	BKV+	未查	阴性	8	正常	MR：右侧半卵圆中心、左侧颞叶及左侧脑室后角旁异常信号，腔隙灶脱髓鞘改变可能	无	+138d	阿昔洛韦丙球	痊愈
13	男	17	MDS	意识障碍、肢体强直、口吐白沫	BKV+	未查	阴性	2	正常	MR：双侧顶枕叶部分皮层下异常信号，应鉴别可逆性后脑病综合征、感染、脱髓鞘改变等	无	+60d	阿昔洛韦	痊愈
14	男	16	MDS	意识丧失、抽搐	BKV+	未查	阳性	0	正常	MR：未见异常	无	+50d	阿昔洛韦丙球	痊愈

注：AML：急性髓系白血病；ALL：急性淋巴细胞白血病；SAA：重型再生障碍性贫血；MDS：骨髓增生异常综合征；CSF：脑脊液；丙球：丙种球蛋白；CMV：巨细胞病毒；EBV：EB病毒

2. 辅助检查：14例患儿中3例脑脊液白细胞增多，4例脑脊液蛋白增高；7例存在影像学改变；4例尿BKV阳性；4例合并CMV、EBV及其他病毒感染。

3. 治疗及转归：3例患儿应用更昔洛韦+丙种球蛋白联合治疗，2例患儿应用阿昔洛韦+丙种球蛋白联合治疗，3例患儿单用更昔洛韦治疗，6例患儿单用阿昔洛韦治疗。其中9例痊愈，5例死亡，病死率为35.7％。

## 讨论

我们回顾性分析2015年1月1日至2020年12月31日于北京大学人民医院接受单倍型HSCT治疗的709例患儿，14例确诊为BKV脑炎的患儿绝大多数为男性（12例），中位年龄为11岁，中位发病时间为移植后第55天，其临床表现不尽相同，最常见的为意识障碍、抽搐发作（7例），其中4例患儿存在出血性膀胱炎，同时尿BKV阳性。14例患儿均采用脑脊液PCR定性方法确诊，其脑脊液常规中，11例患儿未见白细胞增多。14例患儿予阿昔洛韦、更昔洛韦单用，或联合丙种球蛋白治疗，其中1例患儿直接死于病毒性脑炎，4例患儿死于其他疾病，9例患儿痊愈。

BKV是已知的五种多瘤病毒之一，于1971年首次从1例患有输尿管狭窄的肾移植患者中分离出来[11]。BKV感染包括原发感染和重新激活，原发性感染发生在儿童早期，最常见的症状是发热和非特异性上呼吸道感染。在原发性感染发生后，病毒可以继续潜伏于泌尿道上皮细胞、淋巴细胞和可能的其他细胞中。而免疫抑制可导致病毒重新激活，特别是在T细胞缺乏时。BKV感染的相关部位包括肾脏、肺、眼睛、肝脏和大脑，最常见的为肾脏及肺部感染，而中枢神经系统感染很少见[12]。本研究中一部分患者的尿BKV与BKV血症未同时存在，这与Leung等[13]的研究一致：尿BKV阳性和BKV血症之间没有直接相关性，可独立发生。同时本研究中BKV检测为非定量检测，所以不能明确其在血液、脑脊液或尿液中拷贝数是否相关。有研究提出外周血白细胞是BKV的潜伏部位，脑脊液中BKV阳性有可能是白细胞污染导致[14]，或BKV可在体外内皮细胞内复制，从而使BKV通过血液播散至靶器官[15]。还有研究表明，在多发性硬化症、亨廷顿舞蹈症以及没有神经系统症状的患者的脑组织中发现BKV[16]，这表明BKV可能直接潜伏于中枢神经系统中，并在机体免疫抑制情况下被重新激活。本研究中有1例患儿在脑脊液中检测到BKV，但没有病毒血症的证据，也支持这一假设。因我们在无症状患儿中未进行常规的脑脊液BKV测定，所以不能确定是否有脑脊液BKV阳性的无症状患儿。

BKV脑炎通常表现为急性脑炎或脑膜炎的体征和症状[17]–[18]，最常见的症状是头痛及神经损伤，包括癫痫发作、精神症状、定向障碍、幻觉等。在本研究中抽搐发作及精神障碍为BKV脑炎患儿的常见症状，且多数在3个月内发病，因此，当患者存在神经系统症状时，临床医师应充分鉴别药物相关、TMA或脑炎等，提高临床诊断水平。

BKV脑炎的影像学主要表现为脑室周围和脑膜受累[19]，与本报道相符。虽然此影像学改变为非特异性改变，但仍可提示临床医师，在有神经症状或出血性膀胱炎的患者中，影像学提示脑室周围和脑膜受累时需高度怀疑BKV脑炎的可能。

目前还没有统一的BKV中枢神经系统感染的治疗方法，除了减撤免疫抑制剂外，有几种药物已被应用于治疗，如环丙沙星、静脉免疫球蛋白、来氟米特、西多福韦[20]–[23]等，但均效果不佳。本组14例患儿予阿昔洛韦、更昔洛韦单用或联合丙种球蛋白治疗，其中9例痊愈，5例死亡，病死率35.7％，5例死亡患儿中1例死于病毒性脑炎，4例死于严重的GVHD、TMA等其他疾病。虽然减撤免疫抑制剂以恢复免疫有利于病毒的清除，但同样影响了allo-HSCT的成功率，我们需要权衡方案以提高生存率。

在健康个体中，BKV中枢神经系统感染呈现轻度或自限性，通过对症支持治疗即可痊愈。在免疫抑制的患者中，BKV中枢神经系统感染主要表现为脑膜炎或脑炎。虽然我们可以通过患者的脑脊液或脑组织中BKV DNA阳性、影像学表现等确诊BKV中枢神经系统感染，并积极治疗，但结果往往令人沮丧，患者多死于多器官衰竭或其他并发症。因BKV可独立或多部位潜伏于人体器官，且不除外可在内皮细胞内复制，从而通过血液播散至靶器官的可能，所以在allo-HSCT患者中，当患儿有发热、血尿等病情变化时，建议有条件的医院行BKV检测，当患儿有BKV血症、神经系统症状时，必须高度警惕BKV脑炎，尽早行头颅核磁、腰椎穿刺术、脑脊液BKV检测等，明确诊断，尽早给予抗病毒及减撤免疫抑制剂治疗，从而改善HSCT患者的生存及生活质量。
